# Librarian: A quality control tool to analyse sequencing library compositions

**DOI:** 10.12688/f1000research.125325.2

**Published:** 2024-01-24

**Authors:** Kartavya Vashishtha, Caroline Gaud, Simon Andrews, Christel Krueger

**Affiliations:** 1 Independent Researcher, New Delhi, India; 2 Bioinformatics, Babraham Institute, Cambridge, CB22 3AT, UK; 3 Bioinformatics, Altos Labs Cambridge Institute of Science, Cambridge, CB21 6GP, UK

**Keywords:** high throughput sequencing, quality control, sequencing libraries, FastQ, base composition

## Abstract

**Background:**

Robust analysis of DNA sequencing data needs to include a set of quality control steps to ensure that technical bias is kept to a minimum. A metric easily obtained is the frequency of each of the nucleobases for each position across all sequencing reads. Here, we explore the differences in nucleobase compositions of various library types produced by standard experimental methodologies.

**Methods:**

We obtained the compositions of nearly 3000 publicly available datasets and subjected them to Uniform Manifold Approximation and Projection (UMAP) dimensionality reduction for a two-dimensional representation of their composition characteristics.

**Results:**

We find that most library types result in a specific composition profile. We use this to give an estimate of how strongly the composition of a test library resembles the profiles of previously published libraries, and how likely the test sample is to be of a particular type. We introduce Librarian, a user-friendly web application and command line tool which enables checking base compositions of test libraries against known library types.

**Conclusions:**

Library preparation methods strongly influence the per position nucleobase content. By comparing test libraries to a database of previously published library types we can make predictions regarding the library preparation method. Librarian is a user-friendly tool to access this information for quality assurance purposes as discrepancies can flag potential irregularities very early on.

## Introduction

High-throughput sequencing is now a routine technology for the analysis of biological phenomena. A multitude of methods have been developed that obtain genome-wide information on the transcriptome, protein-DNA binding, chromatin compaction, chromosomal conformation and DNA modifications to name but a few. While these approaches address different biological questions and employ various sample preparation techniques, the workflow mostly converges at a stage where adapter flanked short DNA sequences, so called libraries, are subjected to Illumina sequencing. ^1^ The resulting raw data should pass a number of quality control (QC) steps before analysis is performed. ^2^^–^^4^ These can be roughly split into two categories, pre-mapping QC, for example monitoring of base call quality scores, and post-mapping QC, for example overall enrichment scores in ChIP-seq data. For example, raw sequencing data can be queried for adapter contamination and GC bias ^3^^–^^5^ to gauge the quality of the library preparation, or using multi-species alignments to confirm the expected species. ^5^^–^^7^ Early detection of technical biases or problems during sample preparation is important for rigorous data analysis and conservation of resources.

**Figure 1.**
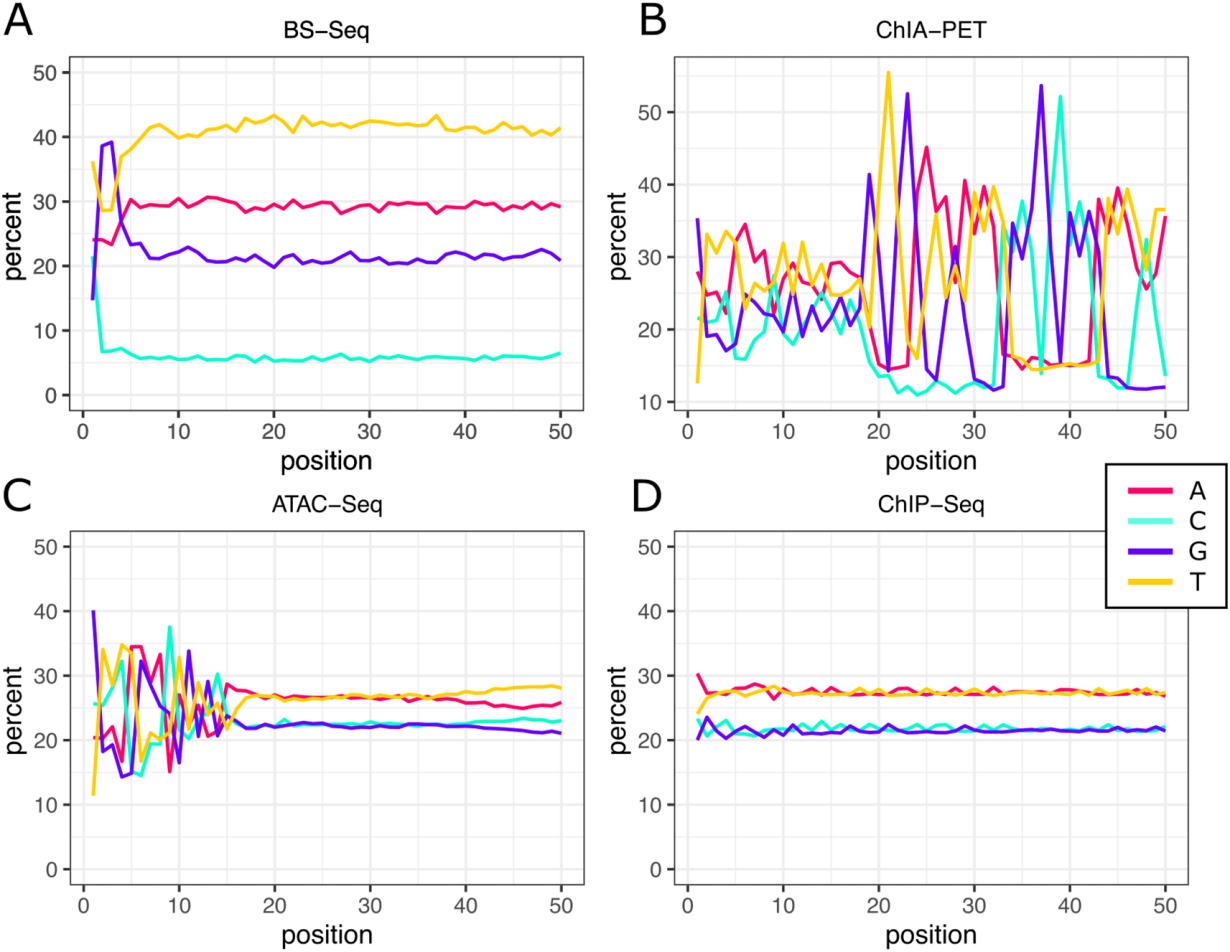
Per position base content for different library types. Base content across the first 50 positions of the sequencing reads was averaged for 436 Bisulfite-seq, 54 ChIA-PET, 416 ATAC-seq and 449 ChIP-seq libraries from mouse and human. Percentages are plotted for each of the four bases. Bisulfite-Seq is used to determine DNA methylation and includes conversion of unmethylated cytosines to thymines which results in the low prevalence of cytosine throughout the read. ChIA-PET is a technology that combines chromatin conformation (Hi-C) analysis with ChIP enrichment. During library preparation a linker is ligated to form paired-end tags which causes the characteristic nucleobase bias in the middle of the read. ATAC-seq is a widely used technique to assess chromatin accessibility and relies on the activity of the Tn5. Target sequence preference of this transposase leads to the observed bias at the start of the read. ChIP-seq allows for enrichment of genomic regions bound by a specific protein which can be pulled down using antibodies. However, as the majority of reads from these experiments originate in non-enriched regions, their base composition mainly reflects the GC content of the organism’s genome.

FastQ is a file format commonly used for storing unmapped sequencing data. One of the metrics that can be obtained from such files is the summarised base composition across the sequencing reads. For each position in the read the respective content of the bases adenine (A), thymine (T), guanosine (G), and cytosine (C) can be determined. For a theoretic random genomic library the expectation would be four horizontal lines reflecting the overall base composition of the genome. Since the GC content of DNA varies according to species ^8^ sequencing libraries will show different composition profiles depending on which organism was sequenced. Less intuitively, libraries produced by different experimental protocols may show vastly different sequence compositions ( Figure 1). A prominent example is Bisulfite-seq ^9^ which is characterised by a strikingly low C content ( Figure 1A). Other library preparation methods like ATAC-seq ^10^ or ChIA-PET ^11^ produce nucleobase bias in specific regions of the read ( Figure 1B, C), while ChIP-seq libraries largely reflect the genome composition ( Figure 1D). Expanding on these observations, we asked if base compositions could be used to distinguish different library types more generally.

The ‘Per base sequence content’ module of the widely used QC tool FastQC ^4^ provides composition information for individual samples, but makes no comparison. Any judgement of whether a particular composition profile is expected for the analysed sample type would require highly specialised niche knowledge which cannot generally be expected of individual researchers. Using the tool MultiQC, ^12^ researchers can collate composition information from multiple individual FastQC reports and visualise them together. This is useful to compare the base compositions of different samples in an experiment and can flag up outliers, but it does not allow for placing samples in the general base composition landscape.

Here, we describe how sample preparation protocols for sequencing libraries result in characteristic composition signatures, and introduce a new quality control tool to check any sequence library against the expected composition of its preparation method.

## Methods

**Figure 2.**
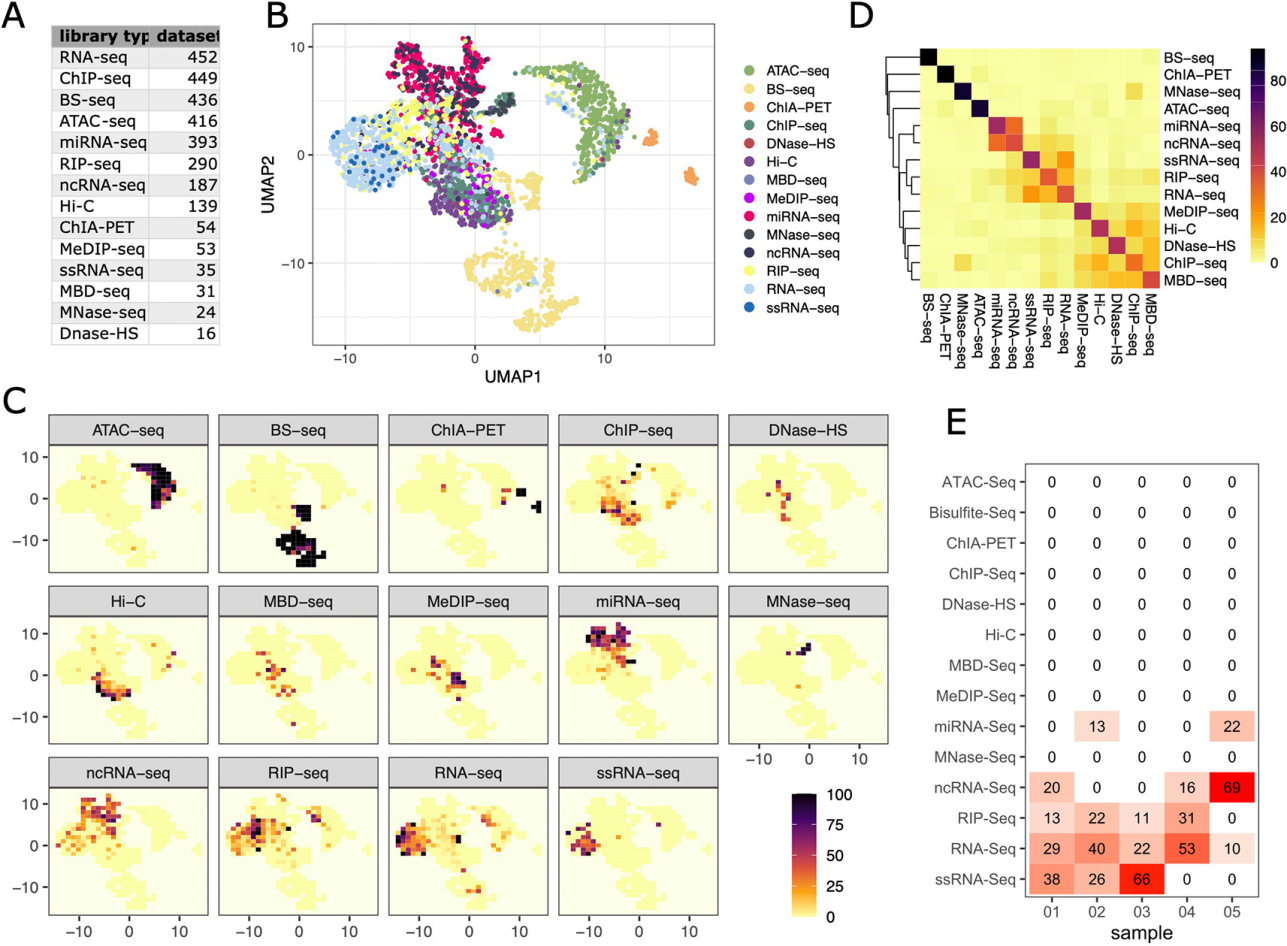
Library types can be distinguished by their base compositions. A) Number of samples per library type included in the analysis. B) UMAP representation of library compositions (reference map). C) Tile based probability map for each library type. Colour represents the percentage of a particular library type per tile. D) Heatmap illustrating the specificity of each library type for tiles of the reference map. All samples were assigned to a reference map tile and colour represents the average percentage of each library type for these tiles. E) Example of Librarian output: Librarian places each test sample in the two-dimensional space of the reference map. This heatmap shows the percent of each library type found in tile associated with the test library.

To get an overview of expected library compositions we queried the open Gene Expression Omnibus (GEO) database ^13^ for high throughput sequencing datasets from mouse and human samples for the years 2018, 2019 and 2020. ^14^ Mice and humans are among the most studied species and are similar in overall GC content (42% and 41%, respectively) making them a good choice to look for compositional differences of different library types. Search results were filtered to exclude library type ‘OTHER’ as well as under-represented types (fewer than 25 samples), and over-represented library types (e. g. ribonucleic acid (RNA)-seq) were capped at 500 samples. Figure 2A shows the number of samples per library type for which per position base compositions could be retrieved.

We then determined how frequently the bases A, T, G and C were found at the first 50 positions in the read (read1 for paired-end data). To visualise sample groupings, the resulting composition data was subjected to Uniform Manifold Approximation and Projection (UMAP) dimensionality reduction ^15^ (using the umap R package with parameters n_neighbors = 15, min_dist = 8) and a two-dimensional representation is shown in Figure 2B (‘reference map’). Interestingly, visually distinct clusters are formed largely along library types, with some library types having very specific base compositions (e.g. Bisulfite-seq, ChIA-PET, ATAC-seq) while others are largely overlapping (e.g. RNA-seq and ssRNA-seq). No systematic difference between mouse and human samples is observed (Supplemental Information ^14^).

To explore how well represented each library type was in each region of the reference map, we split the map into tiles and calculated the percentage of each library type per tile normalised to the total number of samples. Figure 2C shows that, indeed, some tiles are exclusively occupied by a certain library type while others are less specific. To get an overall measure for how well library types could be distinguished, we first annotated each of the samples included in the analysis with its reference map tile. We then averaged the percentages of the library types represented by the tile across all samples of a particular library type to produce the confusion matrix visualised in Figure 2D. While most tiles are very indicative of a certain library type, we also find tiles which are co-occupied by more than one type, for example ncRNA-seq and miRNA-seq. Base composition similarity of certain library types comes as no surprise as the probed material and involved preparation methods can be largely overlapping.

Having concluded that different library types result in largely distinct base compositions along the sequencing reads, we propose to include checking library compositions as a pre-mapping quality control step in the analysis of high throughput sequencing data. This will help flag technical irregularities during sample preparation or potential sample swaps early on and avoid bias during downstream analyses. To make this generally accessible, we developed Librarian ^16^ which allows the user to relate the base composition of any newly sequenced library to other samples in the database.

### Implementation

Librarian will first extract base composition of the first 50 positions of randomly selected 100,000 reads from a supplied FastQ file. It will then project the compositions of the test library onto the manifold created by all libraries in the database as described above, thereby assigning it to a tile on the reference map.

Results are presented graphically: The location of the test sample is indicated both on the reference map and on the plots displaying the probability of each library type per tile. Moreover, the percentages for each library type for the tile assigned to the test library are plotted as a heatmap ( Figure 2E). This lets the user easily gauge how similar the test library is to a collection of published library types.

### Operation

Librarian is available as a web app and a command line tool. In the web app, one or more FastQ files are selected and processed locally to produce the library compositions. Client-side processing avoids upload of large FastQ files and potentially sensitive data. The resulting library composition is compared to the database on the server, and the graphical output can be viewed and downloaded in svg format from the web page.

Librarian can also be run as a command line client application on Linux. Download and install instructions are provided via GitHub (see *Software availability*). Multiple FastQ files can be processed in the same query and summarised output plots are produced. Two modes of cli operation are available: Online query: Just as for the web app, library compositions are compared to the online database to ensure integration of future database expansions with additional library types. Offline: A standalone application is provided which includes the database and allows for analysis in closed environments.

Irrespective of platform, Librarian is only suitable to assess datasets which match the types that the reference map is built on. More specifically, test samples need to have been sequenced with Illumina technology, match any of the included library types and be of mammalian origin (ideally mouse or human).

Documentation for installation and usage, together with FAQs and best practices can be found at https://desmondwillowbrook.github.io/Librarian/.

## Use case

As a use case we assume that a researcher has submitted three samples for sequencing and has now received FastQ files from the provider (use case input ^14^). They want to check if the data conforms to the expectation of the respective library preparation (i. e. RNA-seq, BS-seq and ATAC-seq). Using the Librarian web app, they choose the FastQ files from a directory on their computer and are presented with a graphical representation of how similar their libraries are to published ones regarding their base composition, and a prediction of how likely these samples are to be of a particular library type (use case output ^14^). Any discrepancy to the expected library type should be considered a red flag and investigated further.

Two other use cases could be for a sequencing facility to run Librarian together with other QC packages and provide results to users together with FastQ files as standard. Also, when selecting publicly available datasets for meta-analysis, Librarian can be useful to identify subtypes or biases within the collection.

## Discussion

Our analyses demonstrate that the base composition of sequencing libraries is heavily influenced by the method through which the library was prepared. While this may apply to any sequencing technology, we focussed our efforts on Illumina sequencing as it is by far the most commonly used technology and offers the most diverse applications. Checking the per position base composition can be used as an early quality assurance step for newly sequenced or publicly available data. A sample not matching its expected composition should raise a red flag and the underlying cause should be investigated before moving on with the analysis. While this could point to a sample swap or problem during library preparation, it is also possible that it is caused by a non-standard preparation method.

Of note, within our database of published sequencing libraries we find a small subset of samples which cluster with a different library type. This is nicely illustrated by a group of RNA-seq samples which fall into a region of the map which is otherwise very specific for ATAC-seq. Closer inspection of these examples reveals that their libraries were produced by tagmentation, ^17^ a process that generates short DNA fragments using the same transposase as ATAC-seq. This clearly demonstrates that sequence bias at the start of the read introduced thereby has more of an impact on base composition than the difference between RNA producing genomic regions and generally open chromatin.

We also note that some library types produce distinct subclusters on the reference map. This is particularly obvious for BS-seq libraries. The reason behind this is that BS-seq encompasses a number of distinct library preparation and data processing protocols (e.g. whole genome bisulfite sequencing (WGBS) vs reduced representation bisulfite sequencing (RRBS), or post bisulfite adapter tagging (PBAT) vs non-directional libraries) which produce distinctive base composition profiles. However, only a limited number of metadata tags for library types are available when submitting sequencing data to the GEO database. As sequencing methods diversify, library types grouped by these tags may become more heterogeneous. This illustrates the need to update the library database as new methods are developed and certain commercial library preparation kits change popularity over time. We have therefore built Librarian in a way that can easily incorporate future developments when additional information is shared by the public repository.

## Data availability

### Underlying data

Zenodo: Librarian manuscript data v1, https://doi.org/10.5281/zenodo.10535987. ^14^

This project contains the following underlying data:

- Composition data (output from the original GEO database queries, and datasets included in the Librarian database (filtered list))
- Use case input (example FastQ files (subsampled for smaller file size))
- Use case output (Librarian plots generated from the use case input files)
- Supplementary Information (species differences)

GEO database query parameters: Organism: Mus musculus OR Organism: Homo sapiens AND Platform Technology Type: “high throughput sequencing” AND Publication Date: 2018/010/01 to 2020/12/31.

Data are available under the terms of the GNU General Public License v3.0.

### Software availability

Software available from: https://www.bioinformatics.babraham.ac.uk/librarian/ [Librarian web app]

Source code available from: https://github.com/DesmondWillowbrook/Librarian [Librarian command line download and install instructions]

Archived source code at time of publication: https://doi.org/10.5281/zenodo.10490625. ^16^

Licence: GNU General Public License 3.0

## Author contributions

Kartavya Vashishtha: Software, Writing – Review & Editing

Caroline Gaud: Software, Writing – Review & Editing

Simon R. Andrews: Conceptualization, Funding Acquisition, Software, Writing – Review & Editing

Christel Krueger: Conceptualization, Formal Analysis, Software, Visualization, Writing – Original Draft Preparation
