# Supplementary material for: Librarian: A quality control tool to analyse sequencing library compositions
Source: F1000Res. 2024 Jan 24;11:1122. Originally published 2022 Sep 29. [Version 2] doi: 10.12688/f1000research.125325.2 (PMC9579741; doi:10.12688/f1000research.125325.2)
Supplement: Peer review history

# Reviewer report: Reviewer response for version 2

**DOI:** 10.5256/f1000research.161650.r240420

**Published:** 2024-02-24

**Authors:** Karim Gharbi

**Affiliations:** 1 The Earlham Institute, Norwich, UK

The revised manuscript is significantly improved and addresses the majority of my original comments. The (current) limitations of Librarian have been clarified and accessibility has also been improved.

Are the conclusions about the tool and its performance adequately supported by the findings presented in the article?

Partly

Is the rationale for developing the new software tool clearly explained?

Yes

Is the description of the software tool technically sound?

Yes

Are sufficient details of the code, methods and analysis (if applicable) provided to allow replication of the software development and its use by others?

Yes

Is sufficient information provided to allow interpretation of the expected output datasets and any results generated using the tool?

Partly

Reviewer Expertise:

genomics, next-generation sequencing, bioinformatics

I confirm that I have read this submission and believe that I have an appropriate level of expertise to confirm that it is of an acceptable scientific standard.

# Reviewer report: Reviewer response for version 2

**DOI:** 10.5256/f1000research.161650.r240418

**Published:** 2024-02-15

**Authors:** Andrew Keniry

**Affiliations:** 1 Molecular Medicine Division, Walter and Eliza Hall Institute of Medical Research, Parkville, VIC, Australia

No further comments.

Are the conclusions about the tool and its performance adequately supported by the findings presented in the article?

Yes

Is the rationale for developing the new software tool clearly explained?

Yes

Is the description of the software tool technically sound?

Yes

Are sufficient details of the code, methods and analysis (if applicable) provided to allow replication of the software development and its use by others?

Yes

Is sufficient information provided to allow interpretation of the expected output datasets and any results generated using the tool?

Yes

Reviewer Expertise:

Epigenetics, development, cell biology

I confirm that I have read this submission and believe that I have an appropriate level of expertise to confirm that it is of an acceptable scientific standard.

# Reviewer report: Reviewer response for version 1

**DOI:** 10.5256/f1000research.137618.r151968

**Published:** 2022-10-18

**Authors:** Karim Gharbi

**Affiliations:** 1 The Earlham Institute, Norwich, UK

In this manuscript, Vashishtha et al describes the implementation of a novel quality control (QC) tool for next-generation sequencing (NGS) datasets, which uses nucleotide composition profiles along sequence reads to infer the likely library preparation method used to generate the data. The authors first demonstrate that nucleotide composition is strongly influenced by library method as recorded in the GEO database for a selection of human and mouse NGS datasets. Having established this result, they implemented a program tool (Librarian) to compare library nucleotide composition profiles to a collection of reference datasets and identify libraries with unexpected profiles, which may be indicative of potential failure during library preparation and/or sample/data mix-ups. The tool, which is available as a web application and command line tool, extracts nucleotide composition from user supplied FASTQ files and returns similarity scores against existing profiles stored in the Librarian database. 

 The manuscript is well-written, and the authors provide strong evidence of library method influencing nucleotide composition in the read output files. This will be a familiar observation for those experienced with generating and/or analysing diverse NGS datasets, but the manuscript is a welcome documentation and quantification of these patterns. As a tool, Librarian has the potential to become an important step in the QC of NGS data, alongside other, more generic QC tools, such as FastQC, and help detect quality issues early in data processing. However, I have some concerns about the limitations of the software as currently implemented, which I feel are not sufficiently discussed in the manuscript and could cause significant confusion in the hands of less experienced users. The comments below are intended to help the authors improve the current manuscript and indicate areas for future improvement to increase the usability of the tool. 

**Major comments**

 - Please comment on the applicability of Librarian to data generated with other NGS technologies than Illumina. If not tested or not applicable, this should be highlighted in the discussion. 

 - Please provide a rationale for trimming reads to 50 bases and only considering read 1 to build the database of nucleotide composition profiles, i.e., why is this sufficient to accurately capture the nucleotide composition of each library type. Some methods result in asymmetric library fragments (e.g., 10X Genomics), with different nucleotide compositions in read 1 and read 2, which in itself can be diagnostic of the library type.  

 - The selection of GEO datasets to build reference profiles seems restrictive and potentially biased. Please can you provide evidence that Librarian is applicable to other species than human/mouse, especially species with divergent GC content.

 - The date range filter (01/01/2018 - 31/12/2020) is also likely to have resulted in more recent library types to be excluded from the analysis and therefore the reference dataset. 10X Genomics library types are highly popular, but surprisingly absent. Other library types may have been missed too. 

 - Transposon-based library preparation is increasingly popular and applied across a wide range of library types, including single-cell RNA and DNA sequencing, whole-genome sequencing, ATAC-seq, enrichment capture etc. The authors briefly acknowledge this in the discussion, but it appears to be a major limitation of the tool, i.e., transposon-insertion signature at the start of the reads will likely obscure the underlying library type, causing most transposon-based libraries to cluster together. This should be explicitly documented and investigated further, if possible. 

 - More generally speaking, I would strongly encourage the authors to explicitly identify library types and species "supported" by Librarian, indicating that submission of other library types and/or species may result in inconclusive or potentially misleading results (I acknowledge that the software will accept any FASTQ file).

**Minor comments  **       

 - Please briefly comment on the observed pattern for ChIA-PET and ChIP-seq libraries, i.e., why are these expected and consistent with the library method. ChIA-PET is not a widely used library method. A short description should be included in the text for context. 

 - Please add legend to Figure 1 with key matching coloured lines to individual bases. 

 - I would suggest meta-analysis of public datasets as another important use case for Librarian, e.g., as a clean-up tool prior to meta-analysis or identifying patterns/biases in library type, or subtypes. 

 - Please clarify whether Librarian can we be set up with a local, custom server in addition to query against an online database via the web app or command line tool.

 - The tabular data in figure 2A shows library types with fewer than 25 samples despite these being classified as under-represented libraries and excluded from the analysis in the text. 

 Overall, I believe that the premise of Librarian is a very good idea and thank the authors for their efforts in releasing the program as a publicly available tool. I look forward to reading their responses, and future iterations of the software addressing current limitations.

Are the conclusions about the tool and its performance adequately supported by the findings presented in the article?

Partly

Is the rationale for developing the new software tool clearly explained?

Yes

Is the description of the software tool technically sound?

Yes

Are sufficient details of the code, methods and analysis (if applicable) provided to allow replication of the software development and its use by others?

Yes

Is sufficient information provided to allow interpretation of the expected output datasets and any results generated using the tool?

Partly

Reviewer Expertise:

genomics, next-generation sequencing, bioinformatics

I confirm that I have read this submission and believe that I have an appropriate level of expertise to confirm that it is of an acceptable scientific standard, however I have significant reservations, as outlined above.

# Reviewer report: Reviewer response for version 1

**DOI:** 10.5256/f1000research.137618.r151966

**Published:** 2022-10-14

**Authors:** Konstantin Okonechnikov

**Affiliations:** 1 German Cancer Research Center, Heidelberg, Germany

The manuscript describes the quality control (QC) tool Librarian that predicts the similarity of sequencing library correctness in comparison to the control cohort based on the analysis of reads. Initially for this purpose, the composition of nucleotide variance in the first 50 bp of read was used as input to create a large reference control cohort from publicly available data. Visualisation of these merged nucleotide profiles via UMAP allows to observe clear groups formation based on the data type of a dataset. Novel sample check is a projection into this reference UMAP. Testing the online tool confirmed its usefulness: from inspection of own data, the majority of cases were distinguished correctly. Such projection of a novel dataset into reference would be a useful QC step for any sequencing experiment.  However, the manuscript and the software description could be improved in order to provide more details about the tool as well as explain certain missing blocks.

- In general, the manuscript clearly describes the technique, however, only one possible limitation of the method is stated (effect of a cut in RNA-seq fragments leading to similarity to ATAC-seq in Discussion). More variance factors could be inspected to avoid misleading conclusions about the analysis results. For example, the tissue materials can be obtained from frozen tissue (FFPE), is there any impact of this preparation procedure? In my inspection, the standard RNA-seq datasets were distinguished quite well, but FFPE RNA-seq demonstrated the closest similarity to MBPD and MeDIP-seq. The scRNA-seq protocols are also included, however, they vary since they could be either full gene body covering or only 5’/3’ segment of a gene. Could this have an impact on read nucleotide variance?
- The reads selection is performed with 100K subsampling - how was this amount selected? What is the effect of the total number of reads? Is it sufficient to provide only a subset of them? In this case, what is the suggested limit?
- Also, 50 bp read segment is used as the reference, but how was this selection made? Currently, the main standard for sequencing is 100-150 bp. Would it be more beneficial to use a larger segment of the read for reference generation? Or do quality issues in longer reads have a negative impact?
- How strong is the species effect? Are there variances observed between mice and human materials in full UMAP, e.g. clusters formation? Does it make sense to create own reference for such a procedure, especially when working on other species, e.g. Drosophila?

Further additional comments could help to improve the manuscript for easier reading:

- In Figure 1, the nucleotide type color legend is missing, also segments are not cited in the text directly by suffix (a,b,c,d). Figure 1a demonstrates ChIA-PET, but not clear why it is included since it's not stated in the manuscript text.  
- Figure 2a: Are the amounts of mice and humans mixed? What is the variance?
- Figure 2c: Several enrichment UMAP locations for certain data types are far from each other, e.g. RNA-seq. How to interpret this? Could it be certain subclusters, further splitting the dataset types?
- When downloading example datasets (sample FASTQ files), the archive cannot be opened. Also, there is no documentation available regarding input format preparation, e.g fastq are not allowed to be gzipped, it’s not clear without testing.
- Github documentation on the establishment/launch lacks some details. Would be useful to extend it especially to state what are the system environment requirements before installation.

Are the conclusions about the tool and its performance adequately supported by the findings presented in the article?

Yes

Is the rationale for developing the new software tool clearly explained?

Yes

Is the description of the software tool technically sound?

Yes

Are sufficient details of the code, methods and analysis (if applicable) provided to allow replication of the software development and its use by others?

Partly

Is sufficient information provided to allow interpretation of the expected output datasets and any results generated using the tool?

No

Reviewer Expertise:

Bioinformatics data analysis in pediatric neurooncology

I confirm that I have read this submission and believe that I have an appropriate level of expertise to confirm that it is of an acceptable scientific standard, however I have significant reservations, as outlined above.

# Reviewer report: Reviewer response for version 1

**DOI:** 10.5256/f1000research.137618.r151965

**Published:** 2022-10-06

**Authors:** Andrew Keniry

**Affiliations:** 1 Molecular Medicine Division, Walter and Eliza Hall Institute of Medical Research, Parkville, VIC, Australia

Vashishtha and colleagues perform an analysis of the base composition from ~3000 publicly available sequencing data sets and show that these segregate by library type in a UMAP analysis of fastq files. The authors suggest that this analysis could be a useful pre-mapping QC step to identify incorrect libraries early in analysis pipelines and provide a tool, Librarian, for performing this test. Such an analysis could certainly be useful and has the potential to be widely adopted. I have a few suggestions that could improve the manuscript:

1. The authors show that Librarian identifies libraries prepared by different techniques, but is it able to identify a failed sample within a specific technique? Can the method be tested on failed samples such as ChIP-seq without enrichment, BS-seq with a low conversion efficiency, or samples with high duplication?
2. BS-seq seems to segregate into multiple clusters. Is there an easily identifiable reason for this – perhaps enrichment techniques or developmental stage?
3. I’m not sure of the logistics of this, but Librarian may be more widely used if it was available as an option within the already widely used fastqc.
4. An example of the Librarian output would be beneficial.
5. The terms ‘reference map’ and ‘compositions map’ seem to be used interchangeably. For simplicity, one term should be used throughout.
6. Fig 1A shows the base composition of ChIA-pet data. As this is a less well known technique, it would be beneficial to have an explanation of the base composition results.
7. Fig 1 is missing a legend explaining which base each colour represents.
8. I’m not certain what Fig 2E is showing. Could the authors provide more explanation in the legend? There is also no reference to this figure in the text.

Are the conclusions about the tool and its performance adequately supported by the findings presented in the article?

Yes

Is the rationale for developing the new software tool clearly explained?

Yes

Is the description of the software tool technically sound?

Yes

Are sufficient details of the code, methods and analysis (if applicable) provided to allow replication of the software development and its use by others?

Yes

Is sufficient information provided to allow interpretation of the expected output datasets and any results generated using the tool?

Yes

Reviewer Expertise:

Epigenetics, development, cell biology

I confirm that I have read this submission and believe that I have an appropriate level of expertise to confirm that it is of an acceptable scientific standard.
